# Supplementary material for: Biological Activity and Component Analyses of Chamaecyparis obtusa Leaf Extract: Evaluation of Antiwrinkle and Cell Protection Effects in UVA-Irradiated Cells
Source: Medicina (Kaunas). 2023 Apr 13;59(4):755. doi: 10.3390/medicina59040755 (PMC10146071; doi:10.3390/medicina59040755)
Supplement: Supplementary file 1 [file medicina-59-00755-s001.zip › medicina-2194795-supplementary.pdf]

Supplementary Material

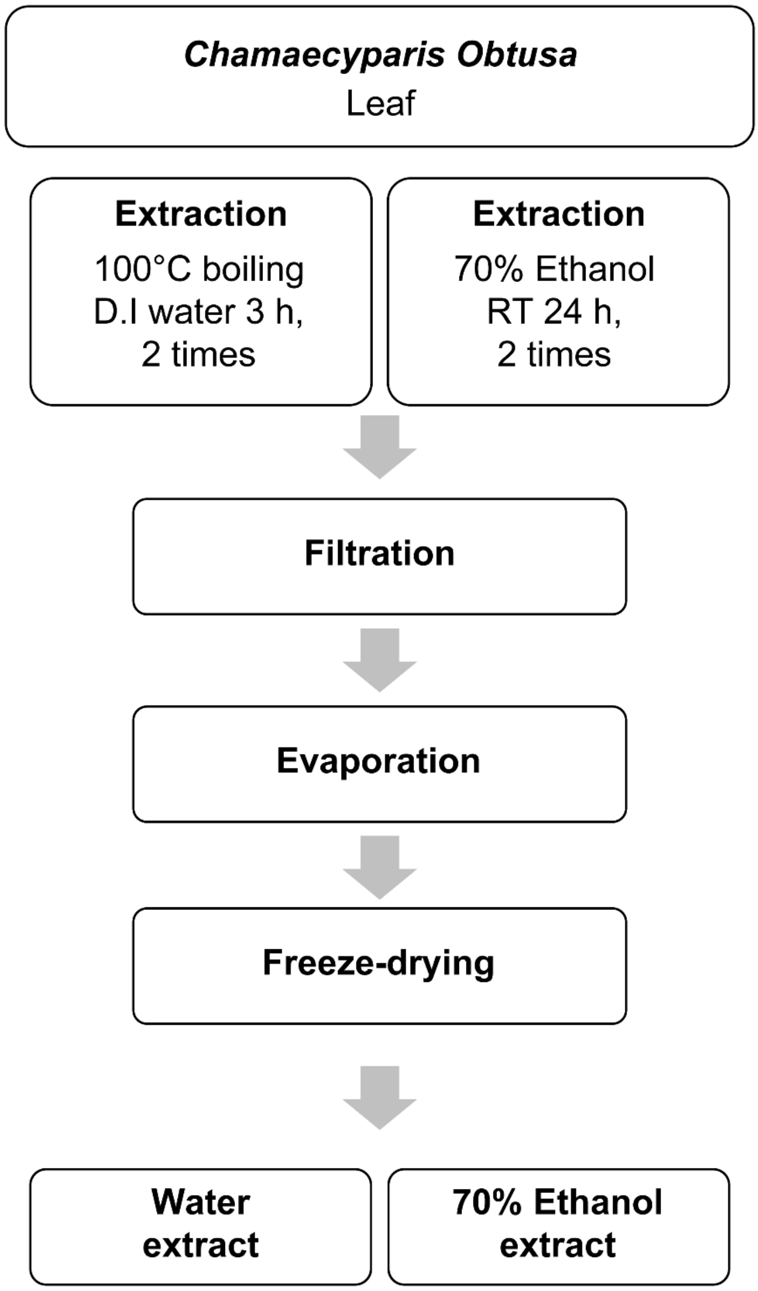

Figure S1: Extraction method of *Chamaecyparis obtusa.*

Table S1: Extraction yield of *Chamaecyparis obtusa.*

Part	Extraction method	Yield (%)
Leaf	Water	19.45%
	70% Ethanol	11.86%

Table S2: Reagents used in this study.

Reagents	Catalog number	Company
2,2-Diphenyl-1-picrylhydrazy (DPPH)	#D9132	Sigma Aldrich (St. Louis, MO, USA)
Tannic acid	#T0200	
L-Ascorbic acid	#A0278	
Folin Ciocalteu's phenol reagent	#47641	
2,2'-Azinobis (3-ethyl benzothiazoline-6-sulfonic acid) (ABTS)	#A1888	
Butylated hydroxyanisole (BHA)	#B1253	
Dimethyl sulfoxide (DMSO)	#472301	
Potassium persulfate ($K_2S_2O_8$)	#216224	
Trizma base	#T6066	
3-(4,5-Dimethyl-thiazol-2-yl)-2,5-diphenyl-tetrazolium bromide (MTT)	#M5655	
1,2,3-Trihydroxybenzene, 2,3-dihydroxyphenol, pyrogallic acid	#P0381	
Xanthine oxidase	#X4875	
Xanthine	#X0626	
Elastase	#E1250	
N-Succinyl-Ala-Ala-Ala-P-nitroanilide	#S4760	
Collagenase	#C0130	
4-Phenylazobenzyloxy-carbonyl-Pro-Leu-Gly-Pro-D-Arg	#89064	
Sodium phosphate dibasic	#1490	Duksan Chemical (Ansan, Korea).
Sodium phosphate dibasic	#1486	
RIPA buffer	# 89900	Gibco BRL Co. (Grand Island, NY, USA)
Dulbecco's modified Eagle's medium (DMEM)	# 11965092	
Fetal bovine serum (FBS),	# 26140079	

Penicillin/streptomycin mixture (P/S)	#10378016	
Hanks' balanced salt solution (HBSS)	#14025076	Sigma Aldrich (St. Louis, MO, USA)
TRI-Solution	#AM9738	Invitrogen (Carlsbad, CA, USA)
cDNA synthesis kit	#PRA5000	Thermo Fisher Scientific, (Waltham, MA, USA)
TB Green® Premix Ex Taq II (Takara Bio, Otsu, Japan).	#RR820A	Takara Bio (Otsu, Japan)

Table S3: High-pressure high-performance liquid chromatography (HPLC) conditions.

HPLC conditions			
Column	Zorbax Eclipse XDB-C18 column		
Column temp.	30°C		
Flow rate	1.0 mL/min		
Wavelength	330 nm		
Injection volume	15 μL		
Mobile solvent	A: Acetonitrile		
	B: Water		
Mobile phase	Time (min)	A (%)	B (%)
	0	10	90
	10	20	80
	15	30	70
	20	70	30
	25	80	20
	30	10	90
	40	10	90
